# Long-Term Assessment of Treatment Timing for Rapid Maxillary Expansion and Facemask Therapy Followed by Fixed Appliances: A Multicenter Retro-Prospective Study

**DOI:** 10.3390/jcm12216930

**Published:** 2023-11-05

**Authors:** Valentina Rutili, Bernardo Quiroga Souki, Michele Nieri, Ana Luiza Farnese Morais Carlos, Chiara Pavoni, Paola Cozza, James A. McNamara, Veronica Giuntini, Lorenzo Franchi

**Affiliations:** 1 Graduate Orthodontic Program, Department of Experimental and Clinical Medicine, The University of Florence, 50121 Florence, Italy; valentina.rutili@unifi.it (V.R.); michelenieri@gmail.com (M.N.); veronica.giuntini@unifi.it (V.G.); 2 Graduate Orthodontic Program, Pontifical Catholic University of Minas Gerais, Belo Horizonte 30535-610, Brazil; bqsouki@gmail.com (B.Q.S.); ana.farnese@icloud.com (A.L.F.M.C.); 3 Department of Faculty of Medicine and Surgery, UniCamillus, International Medical University, 00131 Rome, Italy; dott.chiarapavoni@gmail.com (C.P.); profpaolacozza@gmail.com (P.C.); 4 Department of Orthodontics and Pediatric Dentistry, School of Dentistry and Center for Human Growth and Development, The University of Michigan, Ann Arbor, MI 48109, USA; mcnamara@umich.edu

**Keywords:** Class III malocclusion, cephalometrics, treatment timing

## Abstract

Background: to determine the role of treatment timing in the long-term effects produced by rapid maxillary expansion and facemask therapy (RME/FM) in Class III patients. Methods: This study compared two sample groups treated with RME/FM followed by fixed appliances: the early prepubertal group (EPG) (17 patients; mean age before treatment (T0), 5.8 ± 0.7 years; range, 4.3–6.9 years) and the late prepubertal group (LPG) (17 patients; mean age at T0, 10.1 ± 0.8 years; range, 9.0–11.1 years). Lateral cephalograms for the two groups were examined before treatment (T0) and at a long-term observation (T1) (EPG, 19.8 ± 1.0 years; LPG, 21.0 ± 2.1 years). Independent sample *t*-tests were performed to compare the two groups at T0 and T1. Results: No statistically significant differences were found for any of the cephalometric variables at T0, except for the total mandibular length, overjet, and inclination of the maxillary incisors to the palatal plane, which were greater in the LPG. At T1, no statistically significant differences were detected for any of the cephalometric variables. Conclusions: There were no significant long-term differences when treating Class III patients with RME/FM, either during an early prepubertal phase (≤7 years of age) or during a late prepubertal phase (≥9 years of age).

## 1. Introduction

One of the most common treatment approaches for a Class III malocclusion in growing patients consists of the combination of rapid maxillary expansion and a facemask (RME/FM) [1]. The primary objective of the RME/FM protocol is to enhance the Class III skeletal disharmony by promoting maxillary protraction and controlling the sagittal mandibular position and growth. RME/FM has been shown to achieve good results and a proven efficacy in the correction of this dentoskeletal disharmony in the short term [2], medium term (after puberty) [3,4,5], and long term (end of active growth) [6,7,8,9,10,11]. 

Treatment timing has always been of the utmost importance in the proper management of orthodontic therapy, especially when dealing with a growing patient. The timing of Class III treatment is of fundamental concern, especially because of the multifactorial etiology and the complexity of this disharmony [11]. Furthermore, patients with Class III malocclusion must be monitored for a long period of time (until young adulthood) due to an unfavorable growth pattern [12].

The most appropriate timing to perform RME/FM continues to be debated. Most studies have shown that the best timing is during the early developmental phases (during the deciduous or early mixed dentition) [13,14,15,16]. Chen et al. [17], however, found that treatment for a Class III growing patient would be best accomplished in late mixed–early permanent dentition. Other studies [18,19] stated that there were no differences between treatment with a facemask during early mixed or during late mixed dentition. 

Studies [20,21] on Asian populations indicated no differences in the effects of maxillary protraction treatment performed in a prepubertal or pubertal phase. However, these results are difficult to generalize because different age ranges between groups were considered, and only a few of these studies incorporated indicators of individual skeletal maturity, such as the cervical vertebral maturation [22] or hand-wrist methods [23]. Moreover, most of these studies investigated only the short- or medium-term effect of treatment timing with RME/FM. To our knowledge, there is lack of long-term studies investigating the role of treatment timing using indicators of individual skeletal maturity.

The purpose of this multicenter, retro-prospective study was to assess the role of treatment timing on the long-term effects produced by RME/FM in Class III patients treated during either the early or late prepubertal phases. The null hypothesis is that there is no difference in the long-term intermaxillary sagittal effects produced by RME/FM between the early and the late prepubertal phases.

## 2. Materials and Methods

### 2.1. Study Design

This study was retro-prospective, multicenter, non-commercial (non-profit) and long-term. The study was defined as retro-prospective because the majority of the lateral cephalograms were already available, whereas some of the lateral cephalograms at the long-term follow-up were collected prospectively. The study included data gathered during the period from December 1989 to November 2022.

### 2.2. Settings

Clinical records were screened consecutively to identify patients who fulfilled the eligibility criteria in three centers: the University of “Tor Vergata”, Italy (Center #1), the University of Florence, Italy (Center #2), and the University of Minas Gerais, Belo Horizonte, Brazil (Center #3). Those patients for whom a long-term observation headfilm (at 17 years and older for females and at 20 years and older for males) was not available were recalled prospectively from June 2020 to November 2022.

### 2.3. Participants

The inclusion criteria were:

- Caucasian patients with Class III malocclusion who showed indications for treatment with RME/FM independently from the severity of the dento-skeletal disharmony;
- Prepubertal-to-pubertal stage of maturation at the initiation of therapy (cervical vertebral maturation, CVM stages between CS1 and CS3) [23];
- Availability of lateral cephalogram and panoramic radiograph before the RME/FM therapy (T0). Presence of a lateral cephalogram at a long-term follow up (T1) taken at least at 17 years of age for females and 20 years of age for males.

Regarding patients who were recalled for the T2 record, individuals with an age range between 17 and 25 years were considered.

The exclusion criteria were:

- Patients affected by cleft lip and/or cleft palate;
- Patients with craniofacial syndromes or disorders;
- Patients with congenitally missing or supernumerary teeth;
- For recalled patients, pregnant women were excluded.

Patients in the sample were divided into two groups according to chronological age. In the first group, patients had to have started treatment at an age equal to or younger than 7 years of age, while in the second group, patients had to have started treatment at an age between 9 and 12 years.

### 2.4. Treatment Protocol

#### 2.4.1. Center #1

**Figure 1:**
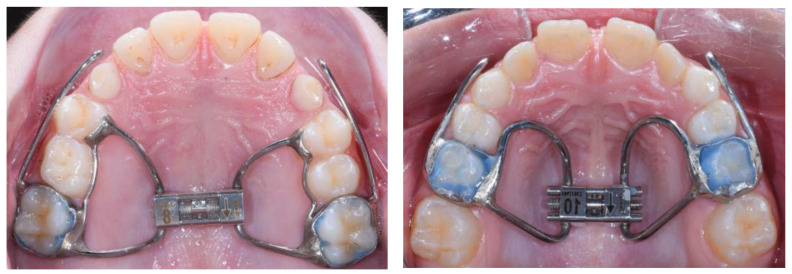
The rapid maxillary expanders used in the 3 centers. On the left, the Butterfly expander and on the right, the Hyrax expander.

**Figure 2:**
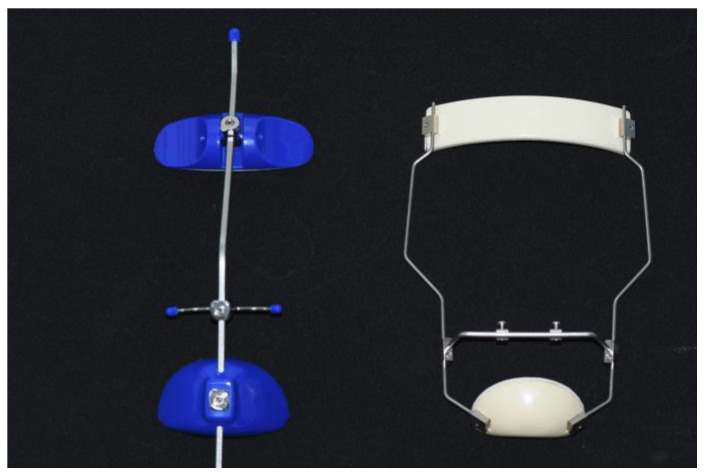
The facemasks used in the 3 centers. On the left, the Petit type and on the right, the Delaire type.

The first phase of treatment consisted of a Butterfly expander [24] (Figure 1) and a Delaire type of FM (Figure 2). A removable bite-block appliance in the mandibular arch was also used in combination with RME/FM treatment. RME was performed with 2 turns per day during the first week, and then 1 turn per day until overcorrection of the transverse discrepancy was achieved.

After the expansion of the maxilla, the amount of expansion was stabilized via locking the expansion screw either with a ligature of 0.012 stainless steel wire or with flow composite, and an FM was given to the patients. Extraoral elastics generating a force of 400–500 g per side were inclined downward at about 30° relative to the occlusal plane.

The mean duration of FM treatment was about 15 months, with the FM that was worn 14 h per day (including nighttime). A retention phase was carried out with a removable mandibular retractor [25] that was worn mainly at night for an average period of 12 months. Fixed appliance therapy in the post-pubertal phase was performed in 80% of the patients. After the second phase of treatment, thermoformed Essix (invisible) retainers in both arches were given to the patients.

#### 2.4.2. Center #2

RME was carried out with either an acrylic splint expander [26] or a Butterfly expander (Figure 1). The expansion screw was activated 1 turn per day until overcorrection of the transverse discrepancy was achieved. After RME, the expander screw was stabilized, and a Petit or Delaire type of FM (Figure 2) was given to patients, with a clinical management protocol similar to Center #1. The mean duration of FM treatment was about 11 months, with an FM that was worn during the night plus as much time as possible during daytime. After the FM, a retention with a removable mandibular retractor was worn for 12–24 months.

Phase 2 post-pubertal treatment with fixed appliances occurred in about 80% of the patients. A retention phase was accomplished in most patients with a Schwarz appliance with Adams clasps on the first permanent molars in both arches or a Schwarz appliance in the upper arch and fixed retainer 3-3 or a thermoformed Essix retainer in both arches.

#### 2.4.3. Center #3

The first phase of treatment consisted of a rapid maxillary expander (Hyrax type) (Figure 1) and a Delaire or Petit type of FM (Figure 2). The expansion protocol was 4 turns on the first day and then 2 turns per day until overcorrection of the transverse discrepancy was achieved. After RME, the expander screw was stabilized, and a Petit or Delaire type FM was given to patients with a clinical management similar to that used at the previous centers. The mean duration of FM treatment was about 12 months with the FM that was worn during the night plus as much time as possible during the day.

The retention protocol was a chin cup worn at night for 6–24 months. Phase 2 treatment with a fixed appliance or aligners was carried out in all patients. A retention phase was performed with a thermoformed Essix worn at night in the upper arch and a 3-3 lingual retainer in the lower arch.

### 2.5. Variables

**Figure 3:**
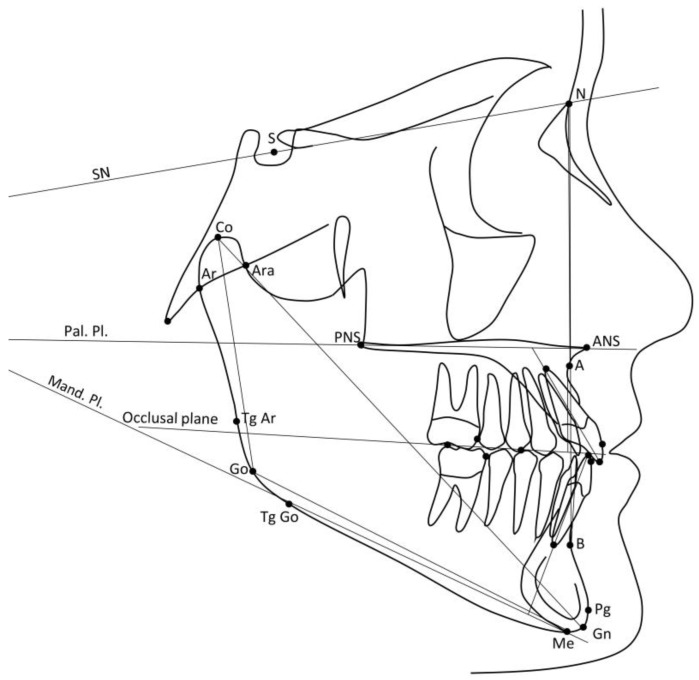
Cephalometric variables. SN line: line between the Sella and Nasion points. Pal. Pl.: line passing through the ANS and PNS points. Occlusal plane: line passing through a contact point on the first permanent molars and a contact point on the first permanent premolars. Mand. Pl.: line passing through the Menton point and tangent to the lower border of the mandible in the gonial region.

The primary outcome variables were the ANB angle and the Wits appraisal [27]. The secondary outcome variables were all the other dentoskeletal cephalometric variables. Digital cephalograms at T0 and T1 were available with a resolution of 150 dpi. All cephalograms were digitized with cephalometric software (Viewbox version 4.1.0.12, dHal Software, Kifissia, Athens, Greece) and they were standardized to 0% magnification (life-size). The 15 cephalometric variables (10 angular, 5 linear) that were measured are illustrated in Figure 3 and described in Table 1.

### 2.6. Methods of Collecting Data

For all patients, the following data were gathered: gender, chronologic age at T0 and T1, and CVM stage [23] at T0. The long-term successful or unsuccessful outcome of comprehensive treatment was evaluated in all patients at T1 following the method described by Souki et al. [28].

### 2.7. Method Error

Twenty cephalograms were randomly selected from the total sample and digitized. The same cephalograms were re-digitized after a wash-out period of 2 weeks by the same operator (V.R.) to check for the intra-observer reproducibility for both the cephalometric variables and the CVM stages.

### 2.8. Bias

Selection bias was reduced by including all patients treated consecutively in the period from December 1989 to November 2022 who complied with the inclusion criteria.

### 2.9. Ethical Permission

Tuscany Region-Central Vast Area Ethics Committee, Florence, Italy (number 16409_oss of 5-5-2020) approved this research. The Ethics Committee verified the compliance of the study with the Good Clinical Practice of the European Union and the ethical principles expressed in the Declaration of Helsinki. For recalled patients, all patients were first informed via phone of the characteristics of the study, and then written informed consent was obtained.

### 2.10. Study Size

To detect a clinically relevant difference in the Wits appraisal of 2 mm with a standard deviation of 2 mm [8], an alpha of 0.05, and a power of 80%, a minimum sample size of 17 patients in each group was required (PS: Power and Sample Size Calculation, version 3.1.6, open source, https://biostat.app.vumc.org/wiki/Main/PowerSampleSize (accessed on 3 January 2020)).

### 2.11. Statistical Methods

Intra-observer reproducibility for the cephalometric variables and for the CVM stages was performed with intraclass correlation coefficients and a weighted K-function, respectively. Descriptive statistics were performed using means and standard deviations for quantitative variables and frequency and percentage for qualitative variables. Independent sample *t*-tests were performed to compare differences between the two groups at baseline (T0) and at the long-term observation (T1). Statistical comparisons for the two dichotomous nominal variables (gender and CVM stage) were performed using Fisher’s exact test. For the comparison of the long-term unsuccessful rate, the odds ratio was calculated. All statistical computations were performed with statistical software (JMP version 13.0.0, SAS Institute Inc., Cary, NC, USA, and MedCalc version 19.6.4, MedCalc Software Ltd., Ostend, Belgium).

## 3. Results

The intraclass correlation coefficient (ICC) was excellent [29] for both the cephalometric variables and the CVM stages.

### 3.1. Participants

**Figure 4:**
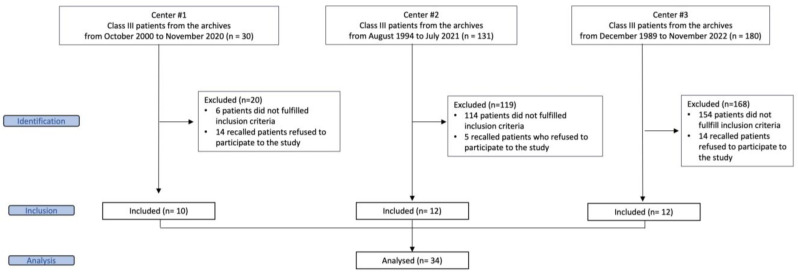
Flowchart of eligible patients.

Figure 4 shows the flow diagram for the assessment of the eligibility of patients. A final sample of 34 patients (22 females and 12 males) was included. All patients at T0 showed a prepubertal phase of development (CS1 or CS2), except for one patient who was in a pubertal phase of development (CS3). Since the two groups included patients who started treatment with RME/FM almost exclusively at a prepubertal phase of development, the first group of patients who started treatment at an age equal to or younger than 7 years of age was named as the early prepubertal group (EPG), while the second group of patients who started treatment at an age between 9 and 12 years was called the late prepubertal group (LPG). Both the EPG and the LPG included 17 patients (EPG: 14 females and 8 males; mean age at T0, 5.8 ± 0.7 years; age range, 4.3–6.9 years. LPG: 8 females and 14 males; mean age at T0, 10.1 ± 0.8 years; age range, 9.0–11.1 years).

### 3.2. Descriptive Data and Main Results

At T0, statistically significant differences were found between the two groups (Table 2). In particular, the LPG showed significantly greater differences in age (4.3 years, 95% confidence interval (CI) from 3.7 to 4.3, *p* < 0.001), total mandibular length (CoGn, 11.3 mm, 95% CI from 8.2 mm to 14.3 mm, *p* < 0.001), overjet (2.7 mm, 95% CI from 1.4 mm to 4.0 mm, *p* < 0.001), and in the inclination of the maxillary incisors with respect to the palatal plane (14.7°, 95% CI from −10.3° to 19.1°, *p* < 0.001).

At the long-term observation, T1 (Table 3), no statistically significant differences between the EPG and the LPG were found for any of the cephalometric variables. Additionally, no statistically significant difference was found between the two groups for the long-term unsuccessful rate (18% in the EPG and 29% in the LPG, odds ratio 1.94, 95% CI from 0.38 to 9.88, *p* = 0.688).

## 4. Discussion

### 4.1. Key Results and Interpretation

This study evaluated the role of treatment timing in patients in the early prepubertal or late prepubertal phase of development treated with RME/FM followed by fixed appliances. This type of approach has important relevance in the decision-making process for growing patients with Class III malocclusion. Although it is still debated, the early treatment of Class III malocclusion in the mixed dentition is recommended strongly for a favorable improvement in Class III dentoskeletal relationships and facial esthetics [30].

The judgment as to whether to treat patients as early as possible (around 4–5 years of age), or at a later stage of growth has been a crucial clinical question debated for the last half century. Delaire [13] asserted that Class III treatment with a facemask is more beneficial if conducted during the deciduous dentition. Most of the studies have demonstrated that the early treatment of Class III malocclusion with an FM during the deciduous or early mixed dentition is more favorable [13,14,15] than treatment at later stages. Contrary to these studies, Chen et al. [17] found that the best treatment timing would be during the late mixed-early permanent dentition. Other studies stated that there was no difference between treatment with an FM during either early mixed or late mixed dentition [18,19] or when treatment with an FM was performed during either a prepubertal or pubertal phase [20,21].

The main limitations of these studies are that different age ranges between groups were considered, and only Cha et al. [21] used an indicator of individual skeletal maturity (the hand-wrist method) to define treatment timing [23]. Moreover, most studies investigated the short-term effect of treatment timing with RME/FM.

Franchi et al. [16], when evaluating the role of treatment timing on the post-pubertal effects produced by RME/FM, found that treatment is most effective when it is started before puberty (early mixed dentition) rather than during the late mixed dentition, with most of the patients treated at puberty. A significant orthopedic advancement of the maxilla that can withstand further maxillary modifications occurring during the active growth period can be achieved only via treating Class III patients before puberty. About 2 mm of supplementary forward movement of the maxilla was maintained in treated patients after the pubertal growth spurt when compared with untreated subjects [16]. Class III subjects treated close to puberty showed a post-pubertal residual amount of 0.7 mm of maxillary advancement versus untreated controls, an amount of growth that is not clinically or statistically significant [16]. This result agreed with the findings of Melsen and Melsen [31] on human autopsy material that showed that the disarticulation of the palatal bone from the pterygoid process is possible only in skulls from the infantile and juvenile prepubertal periods. During these early developmental phases, therefore, the resistance to maxillary protraction is limited, and there is a higher chance of success. On the contrary, when the disarticulation of the palatal bone from the pterygoid process is attempted during the late juvenile and adolescent periods, fracture of the heavily interdigitated osseous surfaces is the most common finding [31]. It should not be surprising, therefore, that RME/FM treatment during the circumpubertal period can result in ineffective maxillary protraction due to the increased sutural resistance [16].

When considering the mandibular changes, Franchi et al. [16] found that both prepubertal- and pubertal-treated groups showed a significant restriction of mandibular growth with respect to the corresponding control groups (3.6 mm in about 7 years, and 4.8 mm in about 4.5 years, respectively). According to these investigators, therefore, the optimal treatment timing for Class III malocclusion with RME and FM is before puberty, when significant favorable modifications in both maxillary and mandibular structures can be achieved. Late treatment, close to puberty, can produce only a significant restriction of mandibular growth.

A crucial clinical question is whether the treatment effects produced by RME/FM are more effective during the early prepubertal phases versus the late prepubertal period. To the best of our knowledge, the current study is the first one that tried to answer this question by evaluating the effects induced by RME/FM in the long term. In this study, significant differences were found between the two groups for three cephalometric variables at T0. This outcome was expected because of the difference in age range between the two groups. The total mandibular length was significantly greater in the LPG (102.8 mm vs. 91.6 mm in the EPG, a difference of 11.3 mm). This result agreed with the results reported in previous studies [19]. This outcome also supports the concept that excessive mandibular growth is a critical aspect involved in the unfavorable growth of this type of malocclusion, particularly in the long term [12].

As for the dentoalveolar variables at baseline, the overjet showed a more favorable value in the LPG (0.2 mm vs. −2.6 mm in the EPG), with a significant difference between the two groups of 2.7 mm. This outcome could be related to the dentoalveolar compensation that occurred due to the significant greater proclination of the maxillary incisors relative to the palatal plane in the LPG (111.3°) relative to the EPG (96.6°).

The present study showed that no significant differences in the long-term observation were found between the two groups. A possible explanation for this lack of differences is that the prepubertal skeletal maturity probably plays a major role in the long-term outcomes produced by RME/FM. The clinical implications of this finding are that there are no differences in the long-term outcomes produced by RME/FM in prepubertal patients treated during either a very early phase (4–7 years) or a later phase (9–11 years). If a patient treated during early prepubertal phases shows early signs of relapse, there is still another chance for a second phase of treatment with RME/FM at a later prepubertal stage. Future studies should focus on the long-term assessment of the role of treatment timing for RME/FM by comparing Class III patients treated before puberty versus Class III patients treated at the pubertal growth spurt.

As for long-term unsuccessful outcomes, the prevalence rate was greater for the LPG (29%) than the EPG (18%), though this difference did not reach the level of statistical significance. This outcome agrees with a previous study by Yuksel and coworkers [19] that reported that the prevalence rate of unsuccessful Class III treatment with RME/FM increased in patients older than 10 years of age. The long-term prevalence rate of unsuccessful outcomes for the early treatment of Class III malocclusion with RME/FM found in this study is consistent with that described in the literature (25–30%) [7,8,20].

### 4.2. Strengths and Limitations

To the best of our knowledge, this is the first long-term study covering until at least 17 years of age in females and at least 20 years of age in males that assessed differences between patients treated with RME/FM in an early prepubertal phase and in a later prepubertal phase. Some heterogeneity was present for the types of appliances used in the three centers (rapid maxillary expander, type of facemask, type of retention). In this study, a control group was not included. However, a comparison with untreated patients with Class III malocclusion was not the aim of this study, as we wanted to investigate the differences between early and late prepubertal treatment. The study was not randomized, even though a randomization with two different timings of intervention is not easy to implement.

## 5. Conclusions

Treatment with the RME/FM protocol in growing Class III patients was equally effective in a long-term observation, whether performed in the early prepubertal or late prepubertal period. The long-term prevalence rate of unsuccessful outcomes was greater for patients treated during the late prepubertal (29%) than during the early prepubertal phase (18%), though it did not reach the level of statistical significance.

## Figures and Tables

**Table 1:** Definitions of the cephalometric variables.

Variable	Definition
NSBa°	Cranial base angle Nasion–Sella–Basion
SNA°	Angle between the SN line and A point
SNB°	Angle between the SN line and B point
ANB°	Angle between the A point, Nasion, and B point
Wits mm	Linear distance between the perpendicular projections of point A and point B on the occlusal plane
SN-Pal. Pl.°	Angle between the SN line and palatal plane (plane passing through the ANS and PNS points)
SN-Mand. Pl.°	Angle between the SN line and the mandibular plane (passing through the Menton point and tangent to the lower border of the mandibular corpus in the gonial region)
Pal. Pl.-Mand. Pl.°	Angle between the palatal plane and mandibular plane
Co-Gn mm	Total mandibular length
CoGoMe°	Mandibular angle
OVJ mm	Horizontal distance measured from the incisal margin of the upper central incisor to the incisal third of the lower central incisor
OVB mm	Vertical distance between the incisal edges of the upper and lower central incisors
Mol. Rel. mm	The distance between the mesial contact heights of contour on the maxillary and mandibular first molars, measured along the occlusal plane.
Upper Inc.-Pal. Pl°	Angle between the long axis of the upper central incisor and the palatal plane
Lower Inc. Mand. Pl.°	Angle between the long axis of the lower central incisor and the mandibular plane

Pal. = palatal; Pl. = plane; Mand. = mandibular; Mol. = molar; Rel. = relationship; Inc. = incisor.

**Table 2:** Descriptive statistics and statistical comparisons between the EPG and the LPG at baseline (T0).

	EPG N = 17 (SD)	LPG N = 17 (SD)	Diff.	95% CI	*p* Value (*t*-Test)
**Age (years)**	5.8 (0.7) Range 4.3–6.9	10.1 (0.8) Range 9.0–11.1	4.3	3.7; 4.3	<0.001
**Females**	14 (82%)	8 (47%)			0.071 (FET)
**CVM** **≥ 3**	0 (0%)	1 (6%)			0.999 (FET)
**NSBa°**	131.3 (6.0)	132.1 (5.5)	0.7	−3.3; 4.8	0.711
**SNA°**	78.7 (3.6)	78.8 (2.7)	0.2	−2.1; 2.4	0.885
**SNB°**	77.9 (3.2)	77.8 (2.7)	−0.2	−2.2; 1.9	0.881
**ANB°**	0.8 (1.9)	1.1 (1.7)	0.3	−1.0; 1.6	0.612
**Wits mm**	−4.6 (4.0)	−4.8 (2.4)	−0.3	−2.6; 2.0	0.815
**SN-Pal. Pl.°**	9.0 (3.3)	9.5 (2.4)	0.5	−1.5; 2.5	0.581
**SN-Mand. Pl.°**	36.1 (4.7)	37.1 (3.5)	1.0	−1.9; 3.9	0.486
**Pal. Pl.-Mand. Pl.°**	27.1 (5.2)	27.6 (3.2)	0.5	−2.5; 3.5	0.476
**Co-Gn mm**	91.6 (4.2)	102.8 (4.5)	11.3	8.2; 14.3	<0.001
**CoGoMe°**	128.3 (5.1)	128.2 (4.0)	−0.1	−3.3; 3.1	0.938
**OVJ mm**	−2.6 (2.0)	0.2 (1.8)	2.7	1.4; 4.0	<0.001
**OVB mm**	0.6 (2.1)	0.9 (1.4)	0.4	−0.9; 1.6	0.565
**Mol. Rel. mm**	2.8 (2.3)	2.2 (1.2)	−0.6	−1.9; 0.7	0.338
**Upper Inc.-Pal. Pl°**	96.6 (5.5)	111.3 (7.0)	14.7	−10.3; 19.1	<0.001
**Lower Inc.-Mand. Pl.°**	83.4 (9.1)	87.3 (5.5)	3.9	−1.4; 9.1	0.145

Diff. = difference; SD = standard deviation; CI = confidence interval; FET = Fisher’s exact test; Pal.= palatal; Pl. = plane; Mand. = mandibular; Mol. = molar; Rel. = relationship; Inc. = incisor.

**Table 3:** Descriptive statistics and statistical comparisons between the EPG and the LPG at the long-term observation (T1).

	EPG N = 17 (SD)	LPG N = 17 (SD)	Diff.	95% CI	*p* Value (*t*-Test)
**Age (years)**	19.8 (1.0) Range 18.4–21.7	21.0 (2.1) Range 17.1–24.4	1.2	0.1; 2.4	0.037
**Unsuccessful**	3 (18%)	5 (29%)	1.94 (Odds ratio)	0.38; 9.88	0.688
**NSBa°**	131.2 (7.6)	131.5 (6.1)	0.3	−4.5; 5.1	0.902
**SNA°**	80.5 (3.4)	80.2 (3.0)	−0.3	−2.5; 2.0	0.798
**SNB°**	79.4 (4.5)	79.4 (3.4)	0.0	−2.8; 2.8	1.0
**ANB°**	1.1 (3.2)	0.8 (2.9)	−0.3	−2.4; 1.9	0.803
**Wits mm**	−3.7 (3.7)	−4.2 (2.6)	−0.6	−2.8; 1.7	0.616
**SN-Pal. Pl.°**	8.8 (3.9)	9.8 (2.6)	1.0	−1.3; 3.3	0.388
**SN-Mand. Pl.°**	32.8 (7.0)	35.0 (5.3)	2.2	−2.1; 6.5	0.303
**Pal. Pl.-Mand. Pl.°**	24.0 (6.8)	25.2 (4.8)	1.2	−2.9; 5.4	0.550
**Co-Gn mm**	115.7 (7.9)	117.5 (6.3)	1.8	−3.2; 6.8	0.464
**CoGoMe°**	122.7 (5.8)	125.0 (5.8)	2.3	−1.8; 6.3	0.264
**OVJ mm**	1.8 (1.5)	1.1 (2.6)	−0.7	−2.2; 0.8	0.348
**OVB mm**	1.5 (1.3)	1.3 (1.7)	−0.2	−1.2; 0.9	0.747
**Mol. Rel. mm**	2.2 (1.9)	3.2 (2.6)	1.0	−0.6; 2.5	0.220
**Upper Inc.-Pal. Pl.°**	116.4 (6.2)	116.8 (4.7)	0.4	−3.4; 4.3	0.826
**Lower Inc.-Mand. Pl.°**	91.0 (9.7)	90.2 (7.6)	−0.8	−6.9; 5.3	0.784

Diff. = difference; SD = standard deviation; CI = confidence interval; Pal.= palatal; Pl. = plane; Mand. = mandibular; Mol. = molar; Rel. = relationship; Inc. = incisor.

## Data Availability

The data will be available on request.
